# Prognostic analysis of surgically treated clear cell sarcoma: an analysis of a rare tumor from a single center

**DOI:** 10.1007/s10147-019-01487-x

**Published:** 2019-06-26

**Authors:** Shiqi Chen, Peng Luo, Lingge Yang, Biqiang Zheng, Zhengwang Sun, Wangjun Yan, Chunmeng Wang

**Affiliations:** 1 Department of Musculoskeletal Oncology, Fudan University Shanghai Cancer Center, 270 Dong’an Road, Xuhui District, Shanghai, 200032 China; 2 Department of Oncology, Shanghai Medical College, Fudan University, Shanghai, 200032 China

**Keywords:** Clear cell sarcoma, Rare tumor, Large sample, Inflammatory biomarker, Neutrophil-to-lymphocyte ratio

## Abstract

**Background:**

The objective of this retrospective study was to evaluate the prognostic value of various factors in clear cell sarcoma patients after radical surgery.

**Methods:**

Forty-two clear cell sarcoma patients from August 2006 to March 2018 were included in the study. Curves of disease-free survival and overall survival were calculated using the Kaplan–Meier method, and univariate and multivariate analyses of various prognostic factors were performed using a Cox proportional hazard regression model. Laboratory test of peripheral blood was recorded before surgery. The optimal cutoff value of systemic inflammatory markers was defined by receiver-operating curve analysis.

**Results:**

The 5-year DFS and 5-year OS rate were 22% and 46%, respectively. The median DFS and OS times were 12 and 41.5 months, respectively. In univariate analysis, there was a significant association between shorter DFS and tumor size larger than 5 cm (*p* = 0.0043), positive surgical margin (*p* = 0.0233), and the neutrophil-to-lymphocyte ratio (NLR) higher than 2.73 (*p* = 0.0009). Furthermore, we observed a significant association between shorter OS and tumor size larger than 5 cm (*p* = 0.0075), positive surgical margin (*p* = 0.0101), NLR higher than 2.73 (*p* = 0.0126), the platelet-to-lymphocyte ratio (PLR) higher than 103.89 (*p* = 0.0147) and the lymphocyte-to-monocyte ratio (LMR) lower than 4.2 (*p* = 0.0445). A multivariate analysis demonstrated that the surgical margin (*p* = 0.013) and NLR (*p* = 0.001) were significantly associated with DFS. Tumor size (*p* = 0.010) and NLR (*p* = 0.013) were independent prognostic factors for OS.

**Conclusions:**

This study had the second largest sample around the world and preoperative NLR may be a useful prognostic factor in CCS patients after radical surgery.

## Introduction

Clear cell sarcoma (CCS), which was first described by Enzinger in 1965 [1], is a rare malignant tumor mainly involving tendons and aponeuroses in young adults. CCS is often misdiagnosed as malignant melanoma due to similar clinical and histological characteristics [2]. Compared with malignant melanoma, CCS often involves a *t*(12;22)(q13;q12) translocation, which leads to a fusion of the activating transcription factor 1 (*ATF1*) gene located at 12q13 and the Ewing sarcoma breakpoint region 1 (*EWSR1*) gene at 22q12, producing the EWSR1-ATF1 fusion protein in a large proportion of patients [3–5].

CCS typically presents as a slow-growing mass with few symptoms, which can lead to a delay in diagnosis and treatment. Furthermore, CCS often spreads rapidly, with quick local recurrence and lymph node and distal metastases already having occurred in many CCS patients at first diagnosis [6, 7]. Currently, the most effective treatment for this tumor type is complete surgical resection, with chemotherapy and radiotherapy mainly serving as palliative therapy [8]. According to other reports, the 5-year OS rate for CCS ranges from 40 to 68% [9, 10].

At present, the role of the inflammatory response in tumorigenesis and tumor progression has been found in recent studies [11, 12]. What is more, systemic inflammatory markers such as neutrophil-to-lymphocyte ratio (NLR), platelet-to-lymphocyte ratio (PLR), lymphocyte-to-monocyte ratio (LMR) have been utilized to evaluate the prognosis of various malignancies [13–16]. However, there was no literature focusing on the correlation between inflammatory markers and the prognosis of CCS. Therefore, we conducted this study to explore the prognostic value of NLR, PLR, and LMR in CCS patients after radical resection.

## Patients and methods

### Patients

Between August 2006 and March 2018, 42 patients with histologically confirmed CCS, who were treated at Fudan University Shanghai Cancer Center, were included in our study. All patients had undergone radical surgery. Clinical information on gender, age, tumor site, tumor size, tumor presentation, microscopic surgical margin classification, important structures (blood vessel, nerve or bone) involved or not, chemotherapy or radiotherapy received was obtained from medical records. With respect to the tumor site, tumor in the armpit, groin, and hip was classified as being in the trunk, and tumor in other sites of the body was classified as being in the extremities. Tumor size information was obtained using specimens or radiographic findings and defined using the longest diameter. The microscopic surgical margin was defined as negative or positive according to the pathology report. Tumor presentation was classified as primary disease and recurrent disease. Adjuvant chemotherapy and radiotherapy were defined when these treatments were used during the time between surgery and the end of follow-up. Laboratory test results such as pretreatment hematologic cell counts were obtained from biochemical examination report before surgery. The NLR was derived by dividing the neutrophil count by the lymphocyte count; the PLR was derived by dividing the platelet count by the lymphocyte count; the LMR was derived by dividing the lymphocyte count by the monocyte count.

### Follow-up data

Patient follow-up occurred every 3 months after treatment for 2 years, followed by every 6 months for the next 3 years, and at 12-month intervals in years 6–15. Follow-up information includes clinical check-up and radiological analysis (ultrasound, computed tomography, or magnetic resonance). Disease-free survival (DFS) was defined as the time between the date of first treatment to the date of disease progression (recurrence, distant metastases, or death). Overall survival (OS) was calculated from the date of treatment to the date of disease-related death. For patients alive and without records of disease relapse, follow-up was censored at the time of the last follow-up.

All histopathological specimens were again reviewed by the Institute of Pathology at the Fudan University Shanghai Cancer Center. This study was approved by the ethics committee of Fudan University Shanghai Cancer Center.

### Statistical analysis

DFS and OS were estimated using the Kaplan–Meier method. According to other reports [9, 10, 17–21], potential prognostic factors such as age, tumor site, tumor size, NLR, PLR, LMR, and surgical margin status were identified using the log-rank test. Differences between groups were compared with the Chi squared test. Univariate and multivariate analyses were performed using a Cox proportional hazard regression model. Factors were included in the multivariate analysis if they were significant in univariate analysis. Receiver-operating characteristic (ROC) analyses were conducted with the OS as an endpoint. The optimal cutoff value of the NLR, PLR, and LMR was estimated at the point of the maximum Youden’s index. The significance level for all statistics was set at *P* < 0.05*.*

## Results

**Fig. 1:**
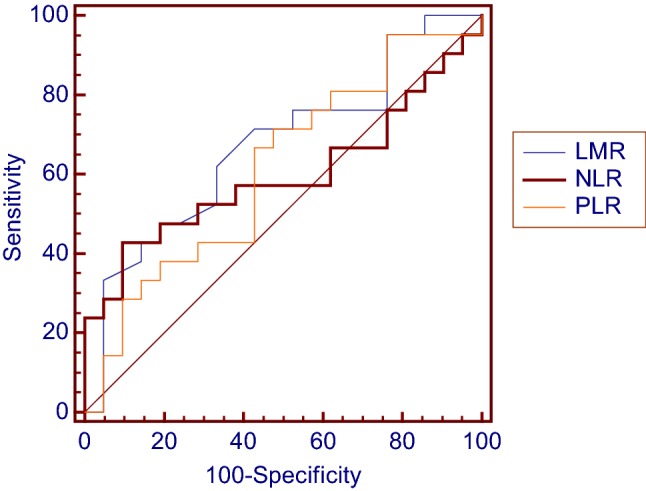
ROC curve analysis of the inflammatory biomarkers in patients with radically resected CCS

Basic information about patients’ characteristics is given in Table 1. The median age of the 23 (54.8%) males and 19 (45.2%) females in the study was 39 years (range 16–74 years old), and the median follow-up time was 45.5 months (range 1.5–146 months). There were 30 (71.4%) patients whose tumors were in the extremities and 12 (28.6%) patients whose tumors were in the trunk. Tumor size of 27 (64.3%) patients were less than 5 cm, with the remaining 15 (35.7%) patients presenting with tumor sizes larger than or equal to 5 cm. The median tumor size was 3.5 cm (range 0.5–15 cm). With respect to tumor presentation, 25 (59.5%) patients presented with primary disease, and 17 (40.5%) patients had recurrent disease. All 42 patients underwent radical surgery, with 34 (81.0%) of them having a negative surgical margin, and 8 (19.0%) patients having a positive surgical margin. The presence of important structures involved at the time of diagnosis occurred in 14 (33.3%) patients, with the remaining 28 (66.7%) patients having none of the important structures involved. Twelve (28.6%) patients received adjuvant chemotherapy and nine (21.4%) patients received adjuvant radiotherapy after surgery. According to the ROC analysis (Fig. 1), the optimal cutoff value of NLR, PLR and LMR was 2.73 [area under the curve (AUC) = 0.596, 95% confidence interval (CI) = 0.434–0.745], 103.89 (AUC = 0.612, 95% CI = 0.450–0.758) and 4.2 (AUC = 0.671, 95% CI = 0.509–0.808), respectively. Details of ROC analysis are summarized in Table 2.

**Table 1:** Patients’ characteristics

Variable	*N* (%)
Gender	
Male	23 (54.8)
Female	19 (45.2)
Age (years)	
≤ 30	14 (33.3)
> 30	28 (66.7)
Tumor site	
Trunk	12 (28.6)
Extremities	30 (71.4)
Tumor size (cm)	
< 5	27 (64.3)
≥ 5	15 (35.7)
Presentation	
Primary	25 (59.5)
Recurrent	17 (40.5)
Surgical margin	
Negative	34 (81.0)
Positive	8 (19.0)
Involving blood vessel, nerve or bone	
No	28 (66.7)
Yes	14 (33.3)
Adjuvant chemotherapy	
No	30 (71.4)
Yes	12 (28.6)
Adjuvant radiotherapy	
No	33 (78.6)
Yes	9 (21.4)
NLR	
≤ 2.73	31 (73.8)
> 2.73	11 (26.2)
LMR	
≤ 4.2	28 (66.7)
> 4.2	14 (33.3)
PLR	
≤ 103.89	17 (40.5)
> 103.89	25 (59.5)

**Table 2:** Results of ROC curve analysis

Variable	AUC	95% CI	*p* value	Maximal Youden’s index	Optimal cutoff
NLR	0.596	0.434–0.745	0.2989	0.3333	2.73
LMR	0.671	0.509–0.808	0.0451	0.2857	4.2
PLR	0.612	0.450–0.758	0.2082	0.2381	103.89

There were 30 (71.4%) patients who suffered from recurrence or metastasis and 9 (30%) of them who were alive at the end of the follow-up period. The median DFS and OS rates were 12 (95% CI = 8.479–15.521 months) and 41.5 months (95% CI = 9.115–73.885 months), respectively. The 5-year DFS and OS rates were 22% and 46%, respectively.

In a univariate analysis, there was a significant association between shorter DFS and the following parameters: tumor sizes larger than 5 cm (median DFS, 7.5 vs. 25.5 months, *p* = 0.0043), positive surgical margin (median DFS, 3.5 vs. 13 months, *p* = 0.0233) and NLR higher than 2.73 (median DFS, 7.5 vs. 25.5, *p* = 0.0009). Furthermore, we observed a significant association between shorter OS and the following parameters: tumor sizes larger than 5 cm (median OS, 23.5 vs. 63 months, *p* = 0.0075), positive surgical margin (median OS, 21.5 vs. 63 months, *p* = 0.0101), NLR higher than 2.73 (median OS, 26 vs. 85 months, *p* = 0.0126), LMR lower than 4.2(median OS, 26 vs. 85 months, *p* = 0.0445) and PLR higher than 103.89 (median OS, 26 vs. 85 months, *p* = 0.0147). Details of the univariate analysis are shown in Table 3.

**Table 3:** Details of univariate analysis

Variable	OS			DFS		
	Median (months)	HR (95% CI)	*p* value	Median (months)	HR (95% CI)	*p* value
Gender						
Male	63	1.217 (0.5169–2.867)	0.6500	10.5	1.694 (0.8234–3.487)	0.1369
Female	36			25.5		
Age (years)						
≤ 30	32.5	1.044 (0.4186–2.604)	0.9253	13	0.8781 (0.4097–1.882)	0.7417
> 30	63			11.5		
Tumor site						
Trunk	23.5	1.226 (0.4776–3.148)	0.6576	12.25	1.198 (0.5466–2.628)	0.6366
Extremities	41.5			12		
Tumor size (cm)						
< 5	63	0.3433 (0.1112–1.06)	0.0075	25.5	0.3786 (0.1606–0.8925)	0.0043
≥ 5	23.5			7.5		
Presentation						
Primary	32.5	1.056 (0.4462–2.498)	0.9010	11.5	0.6944 (0.332–1.452)	0.3113
Recurrent	85			13		
Surgical margin						
Negative	63	0.3175 (0.0821–1.228)	0.0101	13	0.4105 (0.1416–1.19)	0.0233
Positive	21.5			3.5		
Involving blood vessel, nerve or bone						
No	85	0.5535 (0.2072–1.478)	0.1723	13	0.5895 (0.2592–1.341)	0.1504
Yes	36			7.5		
Adjuvant chemotherapy						
No	36	1.831 (0.7137–4.696)	0.1609	4.5	2.051 (0.8328–5.053)	0.0522
Yes	108			14.5		
Adjuvant radiotherapy						
No	41.5	0.6934 (0.2457–1.957)	0.4427	13	0.7526 (0.3149–1.799)	0.4829
Yes	26			9		
NLR						
≤ 2.73	85	0.3509 (0.1224–1.006)	0.0126	25.5	0.3166 (0.1178–0.8513)	0.0009
> 2.73	26			7.5		
LMR						
≤ 4.2	26	2.326 (0.8981–6.023)	0.0445	8.25	1.621 (0.7191–3.652)	0.1851
> 4.2	85			14		
PLR						
≤ 103.89	85	0.3387 (0.1436–0.7992)	0.0147	26	0.5426 (0.2652–1.11)	0.0851
> 103.89	26			11		

**Fig. 2:**
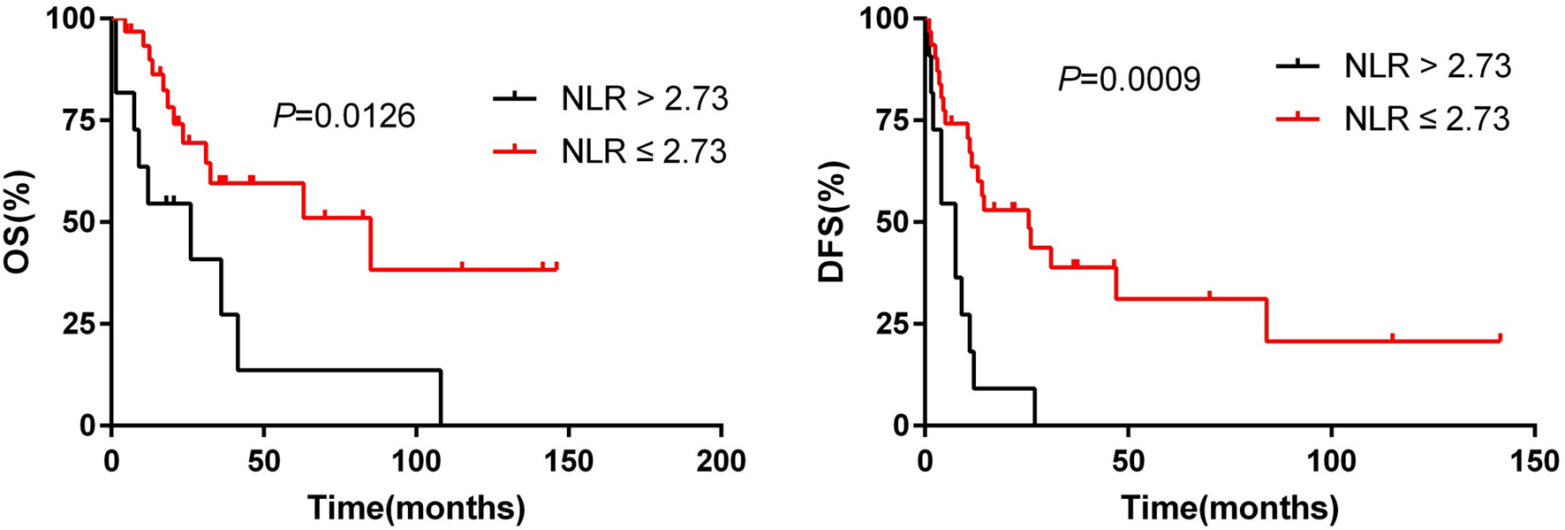
Survival curves according to NLR for OS and DFS

Multivariate analysis (Table 4), performed using a Cox proportional hazard model, demonstrated that surgical margin [hazards ratio (HR) = 2.916, 95% CI = 1.252–6.791, *p* = 0.013] and NLR (HR = 3.992, 95% CI = 1.753–9.093, *p* = 0.001) were significant prognostic factors associated with DFS. Tumor size (HR = 4.214, 95% CI = 1.416–12.538, *p* = 0.010) and NLR (HR = 3.058, 95% CI = 1.264–7.399, *p* = 0.013) were significant prognostic factors associated with OS. Survival curve according to NLR is shown in Fig. 2.

**Table 4:** Details of multivariate analysis

Variable	OS		DFS	
	HR (95% CI)	*p* value	HR (95% CI)	*p* value
Tumor size	4.214 (1.416–12.538)	0.010		0.078
Surgical margin		0.069	2.916(1.252–6.791)	0.013
NLR	3.058 (1.264–7.399)	0.013	3.992(1.753–9.093)	0.001
PLR		0.316		
LMR		0.748

## Discussion

Currently, several researches have revealed that there is a significant association between inflammatory biomarkers and the prognosis of various malignancies including soft tissue sarcoma (STS) [13–16, 21–29]. Nevertheless, STS represents a type of malignant tumor that originates from mesenchymal tissue, which can be classified into different histologic subtypes. In addition, the present studies mainly focused on the correlation between inflammatory biomarkers and STS, lacking subtype-specific research [14, 21, 26, 27, 30]. Moreover, the prognosis of specific histologic subtypes was different, and the treatment strategies varied greatly according to different subtypes. Therefore, we conducted this study aimed to explore the possible prognostic factors and treatment strategies for one of the subtypes of STS–clear cell sarcoma (CCS).

To the best of our knowledge, this is the first study, up to now, focusing on the prognostic value of inflammatory biomarkers in radically resected CCS patients. What is more, our study had the largest sample in the last decade, and it had the second largest sample which only was less than Kawai’s study around the world [9].

In our study, we chiefly assessed the prognostic value of NLR, LMR, and PLR for CCS patients. The most commonly reported studies were that NLR could be a prognostic index for various malignancies including gastric cancers, colorectal cancer, pancreatic cancer, breast cancer and STS [13–16, 25]. In addition, we could also observe a significant effect of NLR on patients’ prognosis including DFS and OS in our cohort. Chan et al. reported that NLR > 2.5 was correlated with worse clinical outcome in STS patients, and NLR > 2.5 was the only independent prognostic factor for OS (*p* = 0.0112) and relapse-free survival (*p* = 0.0125) in multivariate analysis in their study [14]. The slight bias of the optimal cutoff value of NLR could be attributed to the selected patients’ population and the sample size (2.73 vs. 2.5). We only analyzed 42 CCS patients, which are one of the subtypes of STS. While, a total of 712 STS patients were included in the study by Chan et al. As previous reports [13–15, 22, 24], the cutoff value of NLR in various malignancies ranges from 2 to 5, which can be owing to the different pathophysiological characteristics of different tumors. With respect to PLR, one study revealed that decreased PLR was significantly associated with longer OS (*p* = 0.019) and DFS (*p* = 0.032) in multivariate analysis in STS patients [30], while there is only an association between PLR and OS in univariate analysis in our study, and it lost the significance in multivariate analysis. In case of LMR, one previous study that comprised 340 STS patients revealed that low LMR was proven to be significantly associated with decreased cancer-specific survival (CSS) and DFS [31]. However, low LMR was only adversely associated with OS in our study, and it lost the significance in multivariate analysis. The loss of prognostic function of PLR and LMR in our study may be explained by the following reasons: small sample size and the specific pathophysiological characteristics compared to other subtypes of STS.

The solid tumor microenvironment is composed of tumor cells and various immune cells including neutrophils, lymphocytes, natural killer (NK) cells, macrophages and so on [32]. In addition, several types of research have revealed that the interaction between immune cells and tumor cells could promote tumor progression and metastasis [11, 12]. Neutrophils are one of the inflammatory cells which play an important role in tumor growth and metastasis, and numerous reports have manifested that elevated neutrophils are associated with poor prognosis in various tumors including breast cancer, colon cancer, STS and so on [13, 15, 16, 27]. One of the mechanisms of neutrophils in promoting tumor progression is that neutrophils can suppress CD8 + T lymphocyte antitumor response by releasing nitric oxide synthase (iNOS) or arginase 1(ARG1) [33]. Besides, lymphocytes have long been considered to be the main host antitumor immunity, which can inhibit the proliferative and metastatic ability of tumor cells by inducing cytotoxic cell death and cytokine production. In several malignancies, the increased number of tumor-infiltrating lymphocytes was associated with favorable clinical outcome [34, 35]. Moreover, lymphocytopenia is correlated with disease’ severity and the progression of the tumor, indicating a lack of immunologic response to the tumor. Accordingly, elevated neutrophils and reduced lymphocytes can indicate tumor progression and insufficient antitumor immunity, respectively. As a result, we can make the assumption that elevated NLR is correlated with poor prognosis for CCS patients.

Although we have found that NLR is an independent prognostic factor for CCS, our study has certain limitation. For example, we have only 42 patients, although this is already a large sample size for this rare tumor. Multi-center collaboration research is expected in the future to include more patients. Moreover, this study was a retrospective analysis.

**Fig. 3:**
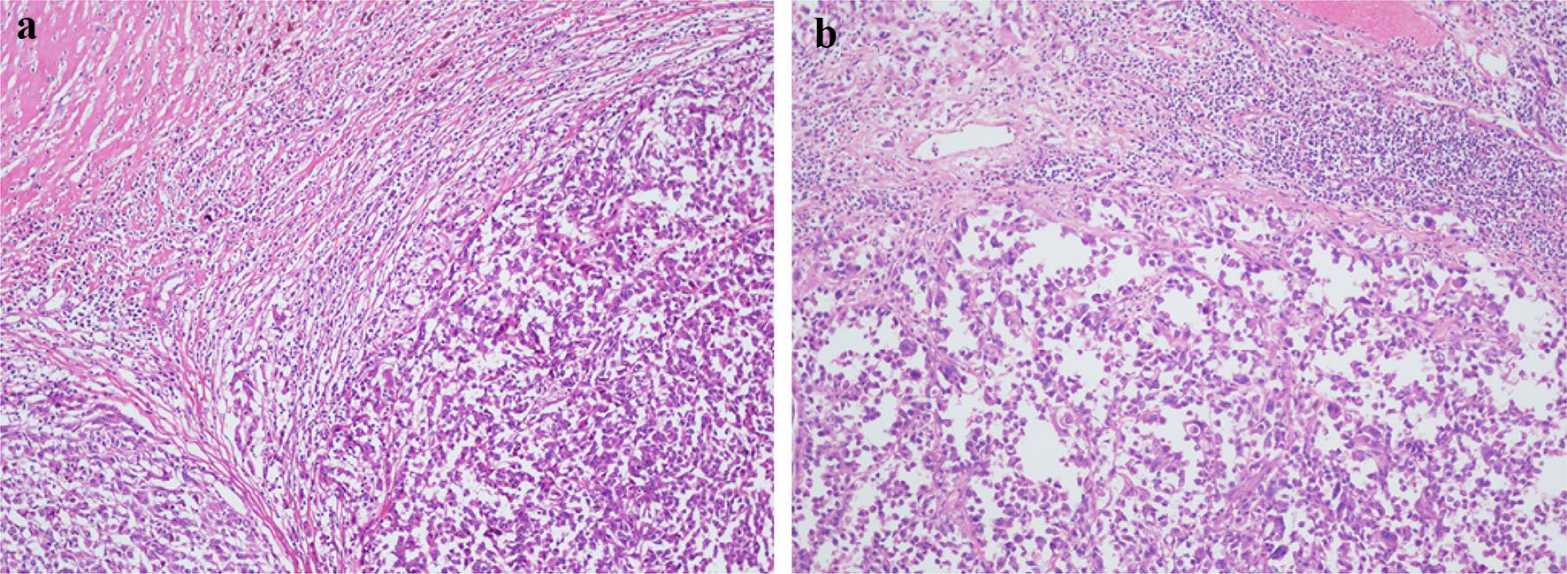
Representative pictures were detected from tumor followed by HE staining for non-specific lymphocytes (magnification × 400)

Currently, CCS is not sensitive to chemotherapy and radiotherapy, and the major treatment remains radical surgery. With respect to the association between peripheral blood NLR and tumor-infiltrating lymphocyte and neutrophil, Dirican et al. [36] found that there was a negative correlation between CD3^+^ TILs number and NLR and a positive correlation between CD5^+^ TILs and NLR. Unfortunately, due to the limited nature of retrospective analysis, we were unable to analyze the association between NLR and tumor-infiltrating lymphocyte and neutrophil. However, we found a large amount of lymphocyte infiltration in the tumor microenvironment by HE staining (Fig. 3), which means that CCS is a hot tumor. Besides, in one case report [37], one patient was found to have achieved complete response after radiotherapy and immunotherapy, because CCS is not sensitive to radiotherapy, this patient may benefit mainly from the treatment of PD1. In general, we hope to provide a new clue whether we can find some drugs that act on neutrophils and lymphocytes for CCS patients through our research since the immunotherapy is developing rapidly.

## Conclusions

To the best of our knowledge, this is the first study, up to now, focused on the prognostic value of inflammatory biomarkers in radically resected CCS patients, which had the second largest sample around the world. Preoperative NLR may be a useful prognostic factor in CCS patients after radical surgery in this study, and this result may provide a rationale for additional studies on CCS.
